# Psychosis as an Evolutionary Adaptive Mechanism to Changing Environments

**DOI:** 10.3389/fpsyt.2018.00237

**Published:** 2018-06-05

**Authors:** Floortje E. Scheepers, Jos de Mul, Frits Boer, Witte J. Hoogendijk

**Affiliations:** ^1^Department of Psychiatry, University Medical Center Utrecht, Utrecht, Netherlands; ^2^Faculty of Philosophy, Erasmus University Rotterdam, Rotterdam, Netherlands; ^3^Department of Child and Adolescent Psychiatry, Academic Medical Center, Amsterdam, Netherlands; ^4^Erasmus Medical Center, Erasmus University Rotterdam, Rotterdam, Netherlands

**Keywords:** psychosis, evolution, adaptation, social exclusion, defense mechanism

## Abstract

**Background:** From an evolutionary perspective it is remarkable that psychotic disorders, mostly occurring during fertile age and decreasing fecundity, maintain in the human population.

**Aim:** To argue the hypothesis that psychotic symptoms may not be viewed as an illness but as an adaptation phenomenon, which can become out of control due to different underlying brain vulnerabilities and external stressors, leading to social exclusion.

**Methods:** A literature study and analysis.

**Results:** Until now, biomedical research has not unravelld the definitive etiology of psychotic disorders. Findings are inconsistent and show non-specific brain anomalies and genetic variation with small effect sizes. However, compelling evidence was found for a relation between psychosis and stressful environmental factors, particularly those influencing social interaction. Psychotic symptoms may be explained as a natural defense mechanism or protective response to stressful environments. This is in line with the fact that psychotic symptoms most often develop during adolescence. In this phase of life, leaving the familiar, and safe home environment and building new social networks is one of the main tasks. This could cause symptoms of “hyperconsciousness” and calls on the capacity for social adaptation.

**Conclusions:** Psychotic symptoms may be considered as an evolutionary maintained phenomenon.Research investigating psychotic disorders may benefit from a focus on underlying general brain vulnerabilities or prevention of social exclusion, instead of psychotic symptoms.

## Background

### Evolution

The past 315.000 years “Homo sapiens” developed and survived all other homo (sub) species. An important characteristic of Homo sapiens is its larger brain size and the capacity of abstract thinking, higher executive functioning and language (1), which created the unique ability of self-reflection, theory of mind, and creativity. However, another, seemingly unique but less advantageous human characteristic is the occurrence of psychotic disorders. During evolutionary development, spreading advantageous genes through reproduction is more important than surviving after the fertile period. One can therefore wonder why genes that make us vulnerable for psychotic disorders, occurring during fertile age, and decreasing fecundity (2), maintained in the population.

### Evolutionary enigma of psychotic disorders

Several hypotheses resolving the “evolutionary enigma” of mental disorders have been described but also doubted [for details see (3–5)]. First, there could be a mismatch between our brain and modern society, similar to the capacity to store energy in fat to survive in times of less food, which in the current Western world can lead to obese people because of the abundance of food. However, psychotic disorders also occur and are as maladaptive in more traditional or pre-modern cultures, which makes the mismatch hypothesis less likely. Second, there could be trade-offs that have net benefits despite substantial costs also called balancing selection. For example, the development of creativity may occur at the cost of mental vulnerability. This balancing selection hypothesis is also not likely since developing alternative adaptive traits nullifying the “costs” of the beneficial features, would logically be favored during human evolution.

A third explanation is polygenetic mutation-selection, suggesting that on-going new mutations in genes with fitness reducing effects on brain function play a prominent role in complex mental disorders. This may be the best explanation for mental disorders with strong reproductive disadvantage, increased risk with increasing parental age, and associations with chromosomal abnormalities. It is stated that many individually rare and evolutionary recent genetic variants reduce mental health, which can lead to disorders like schizophrenia. Although these *de novo* mutations will be pruned out of the genetic pool, an average human being carries over 500 different gene mutations that potentially have fitness reducing effects on brain functioning and even so lead to a continuous distribution of susceptibility. This final hypothesis is not in line with findings of (6) suggesting that there is polygenetic overlap between schizophrenia and genetic signatures of very early human evolution. However, schizophrenia is a complex cluster of different symptoms (psychotic symptoms, cognitive symptoms, negative symptoms…), which could be the result of different pathways in evolution. Maybe genes associated with psychotic symptoms have been in the gene pool for a long time (and thus considered normal) while other genes leading to cognitive vulnerability are continuously changing based on polygenetic mutation-selection. In other words; psychotic symptoms could be a long existing healthy phenomenon in human beings for a specific evolutionary reason while decreased mental capacity with cognitive decline could be the result of multiple different mutations during evolution. Schizophrenia in the core may then be best understood as an a-specific cognitive disorder (instead of a “psychotic disorder”) caused by a broad spectrum of genes that make the brain vulnerable or less adaptive to the environment (5). This leads to a fourth hypothesis that isolated psychotic symptoms should perhaps be considered as a useful mechanism necessary to survive in a specific context (7). This adaptive mechanism occurs in a dysfunctional way only when underlying brain vulnerability leads to a lack of regulation of this mechanism. It is possible that this underlying brain vulnerability is not a fixed given but dependent on the environment as is proposed in the differential susceptibility framework by belsky et al. (8).

In the last two decades, substantial research has been done in the field of psychosis. A wide range of study approaches was used, such as magnetic resonance imaging (MRI), electrical encephalography (EEG), genetics, pharmacology, physiology, neuroendocrinology, and animal models. Despite all this effort, results were sometimes difficult to replicate and most findings were on a population-level with limited individual, clinical relevance (9). Moreover, findings were not always specific for psychotic disorders, which means that multiple genes, EEG/MRI, and physiological changes were also found in other psychiatric disorders (10) and/or (to a lesser extent) in siblings without psychiatric symptoms or healthy controls (11). A direct causal relation between a specific abnormal substrate and psychotic symptoms was never found. If we consider psychotic symptoms as a natural adaptive response to specific circumstances, this is not surprising.

## Psychiatric symptoms as adaptive response, an evolutionary interpretation

Psychiatric disorders are categories of symptom clusters. Isolated symptoms like anxiety, depressed mood and psychosis show a huge overlap between disorders (12). Some of these isolated symptoms are well-known, natural adaptive mechanisms in a specific environmental context. For example, anxiety is a functional mechanism when there is danger and depressed mood can be functional when one has to recover from loss of a beloved one or from physical illness. These symptoms only become “disorders” when they are out of control. This could explain why these “psychiatric genes” have not been eliminated due to natural selection. We simply need the capacity to be anxious or depressed in specific circumstances in the same way we need the capacity of getting a fever to activate the immune system when we are infected with a virus. Eliminating genes that are associated with these defense mechanisms would, from an evolutionary standpoint, be disadvantageous. If we do not run when there is a threat or we do not “down-regulate” in depression after huge physical or mental stress we put our lives or bodies in danger. The extent to which people are responsive/sensitive to the environment may differ for better or for worse (8).

## However, in which circumstances do we need psychotic symptoms?

Psychotic symptoms consist of hallucinations, delusions and distorted behavior. Hallucinations are visual, auditory, olfactory, taste, tactile, or sensory perceptions without equivalent external stimuli, delusions are thoughts and beliefs that are not in accordance with the thoughts and beliefs of others in the “outside” world, and confused or disturbed behavior is behavior that is ineffective and without a goal. From this perspective, psychosis is a state of mind in which reality testing is lacking. However, this psychotic state of mind is much more complex than just a cluster of dysfunctional signs. To lose the border between the inner self and the outside world, for example, we need to have an awareness of the inner world in the first place. In other words we have to have self-consciousness. Self-consciousness is different from consciousness, which also exists in animals and is a relatively simple awareness of the environment, characterized by a first-person (or first-animal) perspective. Self-consciousness, from a philosophical point of view, is the capability of relating eccentric to oneself, being able to reflect on oneself and observe or objectify our own thoughts, behavior and body from a third-person perspective. As (13) states: human beings—modern homo sapiens—not only live (like plants), and in addition experience their lives (like animals) but they are also able to experience their experience (a quality unique to humans and probably to some other higher mammals). Or, to formulate it in terms of embodiment: human beings not only are their bodies (like plants), but they also possess and use their bodies (like animals), and in addition have “out of the body experiences” (13). This may explain the ability of having complex “psychotic” experiences. Being eccentric is both a blessing and a curse. The objectifying stance toward themselves endows human beings with the ability to supplement their life-form with numerous cultural artifacts and practices (which makes them “artificial by nature,” and as such able to adapt to different and changing circumstances quickly), but the complexity of this evolutionary advantage is a fundamental alienation from the world and from themselves: “As an eccentric being, man is not in an equilibrium, he is without a place, he stands outside time in nothingness, he is characterized by a constitutive homelessness. He always still has to become “something” and create an equilibrium for himself” [(13), p. 385; (14)].

Within this perspective, language can be seen as a resource of self-objectification, a tool to place memories in a causal narrative perspective and create new realities. Language plays a crucial role in the conscious awareness of our relationship to the world, to other people in complex social interactions and to ourselves. At the same time it is connected with the conscious awareness of the unbridgeable gap between “the words and the things.” Interestingly, this conscious awareness also seems to play a role in psychosis (15) and the evolution of language and schizophrenia were linked in previous research by Murphy and Benítez-Burraco (16).

Psychotic symptoms occur in almost 17% of the normal population (17). Particularly during adolescence (40–66%) superficial psychotic symptoms can occur without other signs of dysfunction (18). This strengthens the idea that psychotic symptoms itself could be a normal phenomenon based on the described human capacity of conscious awareness.

## Research findings

### Neurotransmitters

What is known is that blocking dopamine receptors in the brain can reduce psychotic symptoms (like painkillers reduce pain), which is one of the most consistent findings in psychosis, although the effect is rather unspecific, has limited/no effect on other symptoms and comes with severe side effects (19). In addition, the balance between the hormones oxytocin (associated with attachment and mother-child bonding) and testosterone (associated with e.g. outgoing behavior), both related to dopamine (20) can be disturbed in psychosis (21) suggesting a relation with social interaction.

### Environment

Although we have argued with Plessner, constitutive homelessness and the accompanied experience of alienation is an integral part of the human condition and we consider psychotic symptoms as a natural protective response, this can become pathological in case of additional factors that induce deregulation. Research focussing on disruptive environmental factors for example revealed an increased risk of psychosis in people with early childhood trauma and/or recent life events (22), after immigration particularly when living in a neighborhood with few others from the culture of origin (23), and when growing up in a high urbanization environment (24). Strikingly, these environmental factors all seem to be related to social exclusion or being alienated from a group. In addition, the relationship between social exclusion and dopamine mechanisms in psychosis was recently reviewed by selten et al. (25).

### Reduced mental fitness

Different cognitive problems that point to reduced fitness of mental functioning are associated with an increased risk of psychosis. For example, lower social skills (26), lower IQ, particularly executive functioning (27), and higher stress sensitivity (28). In addition, insomnia and sleep disorders, which have a negative influence on brain functioning, are associated with hallucinations in the general population (29), and at-risk and first-episode phases of psychosis (30).

## Discussion and conclusions

### Aspects of evolutionary adaptation

In line with the above described research findings, psychosis could be a natural defense mechanism that is necessary to survive as an individual or a group in specific social circumstances [as was also suggested by Cougnard et al. (31)].

Thinking of the most prominent symptoms of psychosis; paranoid delusions, hearing voices, alienating from others, experiencing an increase of significance in details and observations, it seems that psychosis is a complex deregulation of higher abstract thinking with impact on social interaction and the observation of the outside world in relation to one's inner thoughts and feelings. The (self-)conscious mind is in some way “hyper-conscious” (32). The question is; in which life phase can these psychotic symptoms (in a mild or controlled form) be a functional state of mind?

The phase of early development into adulthood lasts relatively long in human beings. From birth to age 4–6 children physically and emotionally depend on their parents and after that, brain maturation takes at least untill age 20–23. In early childhood, children use their parents' emotions to guide and regulate their behavior and to learn about safety and danger in novel situations. This is called social referencing. This social learning is critical for adaptation to the environment (33). From age 6–12 verbal intelligence develops, increasing the capacity of learning facts, basic understanding of language, patterns, and logic. During adolescence particularly non-verbal intelligence and executive functioning of the brain develops, which increases the capacity of abstract thinking, planning, controlling, inhibition, and self-reflection (34). Executive functioning is also associated with social competence (1). The adolescent is preparing for leaving the safe home environment and going out into the world to start an own family. In this new environment one has to find out which places are safe and which are not, which people can be trusted and which not, which signs and signals are important and which can be ignored. To adapt to this new environment the adolescent can only rely on past experiences. Increased observations of signs and detail will trigger earlier experiences, conversations, and feelings in the home environment that were good or bad, positive or negative. One better not trust everyone at first sight, whereas one should also get familiar with new patterns in the surroundings. This phase related situation of being a social outsider takes time and is stressful (35). Being “paranoid,” hyper-alert, feeling different, hypersensitive (36), sleeping less deep with half your brain still being alert in a new surrounding (37), interpreting signs and details as “echoes from the past” and inner observations (38) could be the best way of surviving in this complete new world where one must even find a partner and friends. One better not be naive in trusting people too quickly. “Psychosis” in this perspective could be functional and a natural defense mechanism for leaving home and starting your own life and at the same time decreasing the risk of relating to the wrong (potentially hostile) people. When this natural response to a new environment becomes too extreme or gets out of control, it is not functional anymore. Therefore, controlling or inhibiting the psychotic response is also necessary to return the balance. The control or inhibiting system for psychosis could be the capacity and speed of adaptation to a new environment. To adapt, one needs cognitive flexibility and easy mental shifting, good social skills and competence, and a basic experience of safe and trustful relationships and surroundings (33). Cognitive flexibility, mental shifting, executive functioning, and social skills are found to be disturbed in psychotic disorders (39). This underlying vulnerability may lead to psychotic symptoms that are out of control. Early traumatic or stress experiences, insecure attachment and growing up in the city have a negative effect on basic trust and adaptation to the environment (40). The increased risk of developing psychosis after immigration can also be explained from this perspective, since immigration is an extreme change of environment. When adaptation capacity is not developed fully this may not lead to psychotic disruption in one's own culture but will lead to problems when the change of environment is too immense. In sum, psychosis, as a normal phenomenon, could become problematic when higher cognitive functioning, like executive functioning, social competence, consciousness/self-reflection, and/or abstract thinking are not developed solidly, leading to difficulties in adaptation when someone is in a situation of being a social outsider. The natural defense mechanism is out of control due to a lack of (early developed) adaptation abilities. Every (stressful) change in the environment or hormone/neurotransmitter balance (breaking up a relationship, going to study and leaving home, falling in love, being bullied etc.) leading to a feeling of social exclusion, can trigger the psychotic defense mechanism that is necessary to survive in this strange situation without being able to inhibit it in time by adapting to the new situation properly (41).

### Future directions

Knowing that psychotic symptoms itself are not a pathologic experience, can help people to feel less misunderstood or afraid and could be a way of finding agreement between the patient and the clinician's explanatory model, which will have positive effects on recovery and stigma (42). It will also make the patient-doctor relationship more equal. When psychotic symptoms are explained as a natural (defense) mechanism that is out of control, treatment can focus on the strengthening of the underlying vulnerability and regaining cognitive control instead of curing the psychotic symptoms. Interventions that can train higher cognitive functioning, reduce stress or increase self-consciousness could help people with psychosis to regain control from the inside (43). Antipsychotic medication could help to regulate/inhibit the psychotic defense mechanisms from the outside. The goal of this medication will no longer be to reduce psychotic symptoms to zero, but to decrease them to a level that the patient can live with and deal with during activities. The main focus will be to restore the balance between changes and adaptation in life. Also interventions that focus on making the environment more predictable and less provocative or interventions that stimulate a positive parent-child relationship or increase the social network of a person in order to reduce the effect of social stress could help decrease the risk of deregulation (44). These kind of interventions will increase self-competence and independence (45).

## Author contributions

All authors listed have made a substantial, direct and intellectual contribution to the work, and approved it for publication.

### Conflict of interest statement

The authors declare that the research was conducted in the absence of any commercial or financial relationships that could be construed as a potential conflict of interest.
